# Decoding the Liver-Heart Axis in Cardiometabolic Diseases

**DOI:** 10.1161/CIRCRESAHA.125.325492

**Published:** 2025-05-22

**Authors:** Federico Capone, Antonio Vacca, Guillaume Bidault, Dylan Sarver, Dorota Kaminska, Stefano Strocchi, Antonio Vidal-Puig, Carolina Greco, Aldons J. Lusis, Gabriele G. Schiattarella

**Affiliations:** 1 Translational Approaches in Heart Failure and Cardiometabolic Disease, Max Delbrück Center for Molecular Medicine in the Helmholtz Association (MDC), Berlin, Germany; 2 Department of Medicine (DIMED), Unit of Internal Medicine III, Padua University Hospital, University of Padua, Padova, Italy; 3 Department of Biomedical Sciences, University of Padua, Padova, Italy; 4 Clinica Medica, Department of Medicine, University of Udine, Udine, Italy; 5 University of Cambridge Metabolic Research Laboratories, Wellcome Trust-MRC Institute of Metabolic Science, Cambridge, UK; 6 Department of Medicine, Division of Cardiology, University of California, Los Angeles, CA, USA; 7 Department of Microbiology, Immunology and Molecular Genetics, University of California, Los Angeles, CA, USA; 8 Department of Human Genetics, University of California, Los Angeles, CA, USA; 9 Max Rubner Center for Cardiovascular Metabolic Renal Research (MRC), Deutsches Herzzentrum der Charité (DHZC), Charité -Universitätsmedizin Berlin, Berlin, Germany; 10 Department of Biomedical Sciences, Humanitas University, via Rita Levi Montalcini 4, Pieve Emanuele, 20072 Milan, Italy; 11 IRCCS Humanitas Research Hospital, via Manzoni 56, Rozzano, 20089 Milan, Italy; 12 DZHK (German Centre for Cardiovascular Research), Partner Site Berlin, Berlin, Germany; 13 Friede Springer Cardiovascular Prevention Center at Charité – Universitätsmedizin Berlin, Germany; 14 Experimental and Clinical Research Center (ECRC), a Cooperation of Charité-Universitätsmedizin Berlin and Max Delbruck Center for Molecular Medicine (MDC); 15 Division of Cardiology, Department of Advanced Biomedical Sciences, Federico II University, Naples, Italy; 16 Centro de Investigacion Principe Felipe, Valencia, Spain

**Keywords:** Cardiometabolic diseases, hepatology, cardiology, system biology, HFpEF, MASLD,

## Abstract

The liver and heart are deeply interdependent organs, yet their pathological interplay in cardiometabolic disease has only recently emerged as a key driver of morbidity and mortality. Evidence now positions the liver-heart axis as a central, bidirectional contributor to a spectrum of clinical conditions—including heart failure with preserved (HFpEF) and reduced ejection fraction (HFrEF), atherosclerotic cardiovascular disease, metabolic dysfunction-associated steatotic liver disease (MASLD), alcohol-associated liver disease (ALD), metabolic ALD (MetALD), and cirrhosis.

These diseases do not just coexist – they converge through shared mechanisms such as insulin resistance, chronic inflammation, lipotoxicity, and hemodynamic stress. MASLD, for example, accelerates HFpEF development through myocardial remodeling and diastolic dysfunction, while HFrEF promotes hepatic injury via hypoperfusion and venous congestion. ALD and MetALD amplify cardiovascular risk through oxidative stress, endothelial dysfunction, and prothrombotic states. Cirrhosis leads to cirrhotic cardiomyopathy and arrhythmic complications, even in the absence of overt heart failure. Adding complexity, circadian misalignment exacerbates this interorgan crosstalk, disrupting metabolic homeostasis in both the liver and heart. Secreted factors – including hepatokines (FGF21, FXI, SAA1/4), disrupted metabolite profile, extracellular vesicles and cardiokines (natriuretic peptides, periostin) – mediate this dialogue, linking local pathology to systemic dysfunction.

Deciphering the liver-heart axis requires next-generation research strategies. Preclinical models tailored to MASLD, ALD, and heart failure phenotypes, alongside systems biology, transcriptomic correlation (e.g., QENIE), proximity labeling (e.g., TurboID), organ-on-chip platforms, and precision-cut tissue slices, now offer unprecedented resolution in mapping interorgan signaling.

Understanding liver-heart crosstalk holds the potential to redefine cardiometabolic medicine, identifying novel biomarkers, therapeutic targets, and interventions address the root causes of liver and heart dysfunction in tandem.

## 1 Introduction

Cardiovascular diseases (CVD) are the leading global cause of death driven by complex and multifactorial pathophysiology^1^. Among the contributors to CVD progression, the liver has gained attention as a key player through its complex interplay with the heart. Liver dysfunction has long been considered as separate or secondary to cardiac disease, but growing evidence challenges this view. For example, metabolic dysfunction-associated steatotic liver disease (MASLD), affecting more than 30% of adults^2^, increases cardiovascular event (CVE) risk two-to threefold^3^, making CVD the primary cause of death in these patients. Notably, MASLD often precedes CVD, and this risk persists even after adjusting for concurrent comorbidities^3,4^. Also, alcohol related liver disease (ALD) and later stages of hepatopathy, such as liver cirrhosis, significantly impact on the cardiovascular system. Detrimental effects of liver disease on cardiac function often converge into heart failure (HF), which, at once, may drive congestive hepatopathy and/or liver ischemia in patients with severely compromised cardiac output.

The liver-heart axis involves metabolic, inflammatory, and hemodynamic pathways, with interactions forming a vicious cycle where both organs contribute to disease progression. The majority of these pathways are still under investigation, with a growing body of evidence revealing unexpected mechanisms. Recently, circadian misalignment has been recognized to exacerbate this crosstalk, increasing risk of CVD^5-8^. Disruptions in liver circadian rhythms impair glucose metabolism and lipid regulation, while cardiac circadian dysfunction predisposes individuals to hypertension and increased susceptibility to ischemic heart disease (IHD)^9^.

Investigating liver-heart interactions is complex, requiring advanced research methodologies to validate clinical findings in experimental models. In this review we will examine recent evidence on the liver-heart crosstalk and its impact on CVD. Cutting-edge research techniques for liver-heart crosstalk investigation will be described, moving from multi-organ disease animal models to systems biology approaches, organ-on-a chip and proximity labeling for interorgan signaling. Therapeutic strategies holding promise to break the liver-heart pathological interaction will be described. By integrating current knowledge with innovative research strategies, we aim to equip scientists with tools to explore this axis and uncover its contribution to CVD.

### Physiological Crosstalk Between the Liver and Heart

A stable communication between heart and liver is key for systemic energetic and hemodynamic homeostasis. The liver coordinates blood concentration of glucose, fatty acids (FAs), ketone bodies (KBs) and amino acids. Metabolites produced in the liver are the main energy source for all organs, including the heart, the organ having the highest energy demand per gram of tissue^10,11^. Metabolites supply from the liver to the heart is critical for cardiac energetics and is dynamic. During fasting, glucagon promotes hepatic glycogenolysis and gluconeogenesis, enabling glucose production from non-carbohydrate precursors such as lactate, glycerol, and amino acids^12^. Simultaneously, low insulin levels and activation of the sympathetic nervous system (SNS) promote lipolysis in adipose tissue, releasing FAs into circulation that are subsequently oxidized in the liver and transformed in KBs to be used in the heart^13,14^. Also, during exercise, increased cardiac output (CO) is coordinated with enhanced hepatic gluconeogenesis and higher glucose uptake in skeletal muscles to sustain metabolic balance^15^. SNS activation^16^, cortisol release^17^ and Renin-Angiotensin-Aldosterone System (RAAS) modulation contribute to this highly coordinated liver-heart response^18^.

Conversely, the heart ensures both blood supply to and drainage from the liver, both of which are crucial for its proper function. The liver receives around 1200-1800 ml of blood per minute, representing ¼ of cardiac output and 80% of this volume reaches the organ via the portal vein, collecting blood from the splanchnic region, the body’s largest blood volume reservoir^19^. In normal conditions, the liver and splanchnic vessels serve as a preload reserve, rapidly mobilized to support increased CO. During exercise, stroke volume rises alongside vena cava blood flow. If intrahepatic resistance increases, preload failure may occur, impairing blood return to the heart and limiting exercise capacity.

The liver is also central to lipoprotein metabolism, which facilitates the transport and distribution of lipids throughout the body. The liver synthesizes and secretes very low-density lipoproteins (VLDLs), which deliver triglycerides and cholesterol to peripheral tissues^20^. The liver also prevents lipid accumulation from the bloodstream via chylomicron and lipoprotein receptor-mediated endocytosis and regulating reverse cholesterol transport by high-density lipoprotein (HDL) metabolism. Genetic and non-communicable diseases affecting lipoprotein metabolism in the liver directly promote CVD, as well described in clinical and preclinical settings^21-24^.

The liver serves as the primary organ for protein metabolism^25^, maintaining nitrogen balance (e.g. via urea cycle) and systemic homeostasis. It synthesizes most plasma proteins, including albumin, which regulates oncotic pressure and facilitates the transport of hormones, drugs (including loop diuretics), and FAs. Additionally, the liver produces essential clotting factors necessary for haemostasias^26^. Many are the cardiovascular detrimental effects of deranged protein metabolism in the liver. Altered amino acid metabolism and urea cycle correlate with metabolic dysfunction (e.g. insulin resistance – IR^27^) atherosclerotic cardiovascular disease^28,29^, and HF^30,31^. Low oncotic pressure promotes extracellular fluid accumulation and thus systemic congestion (worsening HF symptoms). Altered coagulation profile, on one hand drives thromboembolic events, on the other hand prolongs clotting time, interacting with antithrombotic therapies. On both sides, thrombotic and hemorrhagic imbalances correlate liver function and CVE. Moreover, several cardiovascular drugs (including direct oral anticoagulants – DOACs, antiarrhythmic drugs, and antiplatelet agents – e.g. clopidogrel) are metabolized in the liver, further entangling, in both a mechanistic and clinical perspective, CVD progression and compromised liver function.

In summary, cardiac and liver functions are highly coordinated. Disruptions in liver-heart axis potentially affect cardiac energetics, exercise capacity, fluid balance, atherosclerotic plaque formation, coagulation profile, and drug metabolism delineating the broad perimeter of liver-heart interaction in CVD.

### A new player in liver-heart axis: circadian rhythm

Beside well-known pathways connecting liver and heart, novel mechanisms have been recently suggested. Regulation of circadian rhythm is among the emerging ones. Circadian rhythms – cycles which exist and repeat across a 24-hour period – optimize fundamental aspects of cellular physiology, such as proliferation, growth, and metabolism, and govern whole-body functions like sleep/wake cycles and feeding behavior. At the core of these 24-hour rhythms is a cell-autonomous molecular oscillator present in every cell. The molecular clock consists of core clock components that generate 24-hours rhythms in the expression of clock genes (Bmal1/Arntl, Clock, Cry1/2, Per1/2, and Nr1d1/2) and clock-output genes via coupled transcriptional and translational feedback loops^32,33^. Correct timing of these molecular clocks relies on entrainment factors called zeitgebers (time givers). For the central clock in the suprachiasmatic nucleus (SCN), light is the primary zeitgeber, while peripheral clocks in other tissues are entrained by neuronal, endocrine, and feeding-related signals^34-38^. Disruptions to circadian rhythms—caused by chronic jet lag, shift work, or genetic alterations—are linked to a range of cardiometabolic disorders, including T2D, obesity, metabolic liver diseases, and HF^5-8^. Conversely, strategies like time-restricted eating (TRE) and light therapy can strengthen circadian rhythms and improve cardiometabolic health^39,40^.

As a central processor of whole-body metabolism, the liver is profoundly influenced by circadian rhythms^7^. Approximately 15% of the liver’s transcripts exhibit diurnal oscillations, with peaks timed to feeding and fasting cycles^41-43^. These oscillations regulate critical metabolic processes, including glycogenesis, glycogenolysis, gluconeogenesis, lipogenesis, and fatty acid oxidation, enabling the liver to anticipate and respond to nutrient availability. Glycogenesis and lipogenesis in the liver are driven by rhythmic activation of enzymes such as glycogen synthase (GYS1/2)^44,45^ and of the lipogenic transcription factor SREBP1c^46,47^. Core clock genes tightly regulate also catabolic pathways activated during fasting. For example, CRY proteins repress gluconeogenic enzymes^48^ and PERIOD proteins regulate the circadian expression of mitochondrial enzymes to drive daily rhythms in mitochondrial respiration^49^. Hormonal signals like insulin and glucagon further synchronize hepatic rhythms with nutrient availability, ensuring a balance between energy storage and mobilization^50,51^. Disruption of these rhythms–through irregular eating patterns or genetic mutations in clock genes—can lead to metabolic imbalances, increasing the risk of liver disease. For instance, liver-specific deletion of Bmal1 impairs glucose homeostasis, promoting IR and steatotic liver disease^52^. Additionally, the gut microbiome and its oscillatory species, and the metabolites they produce play a role in regulating hepatic metabolic homeostasis^53,54^.

The heart, similar to the liver, operates under precise circadian regulation^55^. Unlike the liver, however, the heart relies primarily on oxidative metabolism, with its fuel preferences shifting between glucose and FAs across the day. During the active phase, the heart preferentially uses glucose, while FAs dominate during the rest phase. These shifts align with nutrient availability and metabolic activity in other tissues. The cell-autonomous clock of cardiomyocytes orchestrates these metabolic rhythms by regulating the expression of enzymes involved in glucose uptake, glycolysis, mitochondrial oxidative phosphorylation and lipid homeostasis. For example, BMAL1 enhances myocardial glucose oxidation during the active phase^56^, while REV-ERBs modulate fatty acid oxidation^57^. Disruption of these rhythms -through shift work or sleep deprivation – impairs myocardial energy homeostasis, increases oxidative stress, and contributes to CVD. Studies in mice show that deleting core clock genes (Bmal1, Per1/2, Rev-erba/b) in the heart leads to impaired contractility, hypertrophic remodeling, HF, and increased susceptibility to IHD^9^.

Circadian regulations extend beyond energetics. For example, hepatic enzymes for xenobiotic detoxification peak during rest, while those for glucose and lipid metabolism peak during activity^58,59^.

Together, these findings underscore the intricate role of circadian rhythms in coordinating liver and heart function, extending beyond energy metabolism to include diverse physiological processes. Disruption of these rhythms can compromise organ-specific and systemic homeostasis, highlighting circadian alignment as a potential therapeutic target in liver-heart axis disorders.

## 2 Liver diseases affecting cardiovascular system

### MASLD: a key accelerator of cardiometabolic syndromes

Disruptions in liver-heart cross-talk contribute to the pathogenesis of a number of CVD, but the subgroup of cardiometabolic disorders are of particular interest and concern. Cardiometabolic diseases are interrelated disorders including obesity, T2D, dyslipidemia and hypertension, which frequently coexist and synergistically drive CVD such as IHD, cerebrovascular disease, and HF. Cardiometabolic disease are a massive threat to global health. Almost half of the adults aged 25 or older are living with obesity^60^ and T2D affects more than 500 million people worldwide, a prevalence expected to double by 2050^61^. In 2021, 3.71 million deaths were attributable to overweight and obesity^62^ and most of these deaths were due to CVD.

MASLD is a highly prevalent cardiometabolic disease. For long been considered as an exclusion diagnosis in the evaluation of patients with lipid accumulation in the liver, in absence of significant alcohol intake (non-alcoholic fatty liver disease), MASLD is now univocally recognized as a metabolic disease, affecting more than 30% of adults globally ^63^, with a prevalence rising to 55-70% in patients with T2D ^64-66^ and 70-75% in overweight or obese individuals ^67^. MASLD is defined by the presence of steatotic liver disease (SLD, defined as ≥5% liver fat content confirmed by imaging, biopsy, or biomarker-based assessment) in combination with at least one out of five features of metabolic syndrome (overweight or obesity, dysglycaemia or T2D, high plasma triglycerides, low HDL cholesterol, hypertension) and no other discernible cause for SLD ^2^. The spectrum of MALSD span from milder forms with little or no inflammation to metabolic dysfunction-associated steatohepatitis (MASH), further progressing to liver fibrosis, cirrhosis and MASH-related hepatocellular carcinoma (HCC) in most severe cases ^2^. Although the precise prevalence of MASH remains less clear, current evidence indicate 2% to 6% of the global adult population to be affected^68^. Notably, this risk is considerably higher in individuals with T2D, with MASH identified in 37% of this population and advanced fibrosis observed in 17%^64^. Similar trends are seen in obese individuals, where 33% have MASH and 7% present with advanced liver fibrosis^67^. Progression to cirrhosis is observed in 20% of MASLD patients, with fibrosis severity serving as a key predictor of adverse liver related events^69^. Additionally, T2D further elevates the risk of hepatic decompensation and hepatocellular carcinoma in these individuals^70^.

Considering the rising prevalence in obesity and T2D, the number of MASLD related HCC and liver transplants is expected to double and quadruple respectively by 2050 in the US^71^.

### The impact of MASLD on cardiovascular risk: epidemiological data

Increase in liver-related events, however, is not the major threat in MASLD. Subjects with MASLD have significant higher risk of CVE^72,73^, with CVD representing the leading cause of morbidity and mortality in these patients^74-76^. While some reports indicate liver-related events as the main cause of death in MASLD^77^,other evidence confirm that CVD and cancer are more prominent contributors to mortality^63^.

Robust epidemiological data indicate MASLD contribution to increased risk of CVD to be independent of coexisting risk factors in affected patients. In a Swedish cohort, MASLD independently increased the incidence of CVE, including IHD, stroke, HF, and cardiovascular mortality, with the risk escalating as the severity of MASLD progresses^78^. A retrospective study including over 111,000 patients, further revealed that MASLD was associated with a 1.54 times higher incidence of acute myocardial infarction (AMI), independently of other risk factors^79^.

MASLD is specifically correlated to one HF phenotype, namely heart failure with preserve ejection fraction (HFpEF)^80^. MASLD is present in up to 50% of HFpEF patients, with advanced liver fibrosis found in 8-38%, and cirrhosis in 7-12%^81-83^.

MASLD correlates with several aspects of diastolic dysfunction, including increased LV mass, decreased E/A ratio and increased E/e′ ratio, elevated LV filling pressure, reduced global longitudinal strain^84,85^ and increased left atrial volume^86,87^. This correlation is independent of age, sex, obesity, hypertension, and T2D ^78,80,86-94^. Consistently, advanced MASLD stages, particularly those with hepatic fibrosis, are linked to a higher prevalence of diastolic dysfunction^81,94-98^ and predicts worse HF outcomes and higher mortality^82,99,100^.

However, altered cardiac function is also present in the earlier stages of MASLD, as evidenced by subclinical structural and functional changes in asymptomatic MASLD patients that increase susceptibility to diastolic alteration and HFpEF, independent of other cardiometabolic risk factors^101,102^. Another finding supporting that coexistence of MASLD and HFpEF is not only explained by shared risk factors is that lean patients with NAFLD, with favorable metabolic profiles, still exhibit an increased risk of cardiometabolic disease and mortality^103^, an elevated risk at least partially mediated by cardiac remodeling and LV diastolic dysfunction^104,105^.

MASLD is also intricately tied to atherogenic dyslipidemia and hypertension, both of which amplify CVD risk through interconnected metabolic and inflammatory pathways^106^. A recent meta-analysis involving Western and Asian cohorts demonstrated that MASLD is associated with increased carotid intima-media thickness (OR 2.00, 95% CI: 1.56–2.56) and a higher prevalence of coronary artery calcification (OR 1.21, 95% CI: 1.12–1.32)^107^. Notably, this association persists in individuals with severe coronary artery calcifications^107^.

However, despite MASLD’s strong association with increased atherosclerotic cardiovascular diseases (ASCVD), this risk does not appear to independently translate into increased ASCVD mortality after accounting for traditional cardiovascular risk factors. Two recent meta-analyses concluded that while MASLD increases ASCVD risk, the association with ASCVD mortality becomes non-significant after adjusting for factors such as age, sex, and comorbidities^108,109^. Some evidence suggests that advanced MASLD stages, particularly those with higher fibrosis scores, may pose a greater risk for cardiovascular outcomes^110^. However, a prospective study involving biopsy-confirmed MASLD patients found no apparent difference in cardiac event rates based on fibrosis stages^69^.

### Pathophysiological underpinnings connecting MASLD and CVD

A complex pathophysiology links MASLD to the progression of CVD disease. IR is key in MASLD progression and significantly contributes to CVD. Adipose tissue IR prevent the suppression of postprandial lipolysis, elevating FA delivery to the liver in the fed state. Simultaneously, it enhances hepatic *de novo* lipogenesis and impairs fatty acid oxidation, both contributing to liver steatosis^111^. Hepatic IR also leads to increased gluconeogenesis—a process normally suppressed by insulin—resulting in hyperglycaemia. Elevated blood glucose is a well-established contributor to endothelial dysfunction and oxidative stress (PMID: 21030723, PMID: 21747057) which are implicated in the development of both ASCVD and HF (PMID: 16618833, PMID: 23684677). Liver-induced hyperglycaemia also promotes the formation of advanced glycation end-products (AGEs), which contribute to oxidative stress and myocardial collagen crosslinking, leading to fibrosis and impaired ventricular compliance, which are hallmarks of HFpEF (PMID: 18071071; PMID: 26678809). Importantly, not only IR drives MASLD but the opposite is also true. Lipid accumulation in the liver activates cytokine release (including mediators such as fibroblast growth factor 21 - FGF21, fetuin A and B), inflammatory pathways and altered lipoprotein metabolism, all affecting metabolic balance in pancreas, adipose tissue and skeletal muscle and thus promoting glucose intolerance^112^. This vicious cycle exacerbates IR severity, in turn driving CVD. A 2012 meta-analysis involving over half a million participants demonstrated that IR significantly elevates ASCVD risk, with a one standard deviation increase in the Homeostasis Model Assessment for Insulin Resistance (HOMA-IR) index correlating with a 1.46-fold higher risk of developing ASCVD^113^. Similarly, HOMA-IR scores have been independently associated with altered LV relaxation across heterogeneous clinical cohorts, with altered diastolic dysfunction estimated to affect up to 50% of T2D patients^114-116^. IR contributes to ASCVD and HF via several mechanisms. In healthy individuals, insulin exerts vasodilatory and anti-inflammatory effects by stimulating nitric oxide (NO) production via the phosphoinositide 3-kinase (PI3K)/Akt pathway^117^. However, in the context of IR, there is an imbalance in insulin signaling, with impaired PI3K/Akt activation alongside compensatory overactivation of the mitogen-activated protein kinase (MAPK) pathway. This shift promotes vasoconstriction, endothelial dysfunction, and inflammation — all of which contribute to atherosclerosis development and diastolic dysfunction^118,119^. Moreover, IR affects cellular lipid handling in the myocardium. Specifically, IR promotes the preferential positioning of CD36 on the sarcolemma and induces GLUT4 internalization, enhancing lipid uptake and intracellular lipid accumulation. This lipid overload may exacerbate myocardial dysfunction through lipotoxicity, a mechanism particularly relevant in the development of HFpEF^120-122^.

The altered lipid profile of MASLD, marked by elevated triglycerides, reduced HDL-C, increased LDL particles, and elevated total cholesterol, also contribute to the increased risk of ASCVD in these patients^123-125^. Notably, experimental evidence demonstrates that hepatic IR alone is sufficient to drive atherogenic dyslipidaemia in mice, thereby establishing a direct mechanistic link between MASLD and ASCVD (PMID: 18249172). Concurrently, IR and chronic inflammation drive hypertension by increasing SNS activity, vasoconstriction, and blood volume, necessitating integrated management to mitigate these compounded effects^126,127^. Elevated circulating triglycerides and FAs in MASLD can lead to ectopic lipid accumulation in non-adipose tissues, including the heart. In the context of hepatic IR, liver-induced hyperglycaemia inhibits myocardial lipid oxidation thus exacerbating myocardial lipid accumulation (PMID: 9497163). Myocardial lipid accumulation is positively associated with diastolic dysfunction, independently of classical risk factors (PMID: 31696627, PMID: 19022158), and cardiac steatosis positively correlates with intrahepatic fat content, suggesting systemic lipid spillover (PMID: 19022158). Thus, this excess lipid deposition contributes to cardiac lipotoxicity, disrupting myocardial function and increasing the risk of HFpEF^128,129^. Moreover, MASLD elevates the risk of CVE by promoting a hypercoagulable state through increased levels of coagulation factors, impaired fibrinolysis, enhanced platelet reactivity, and endothelial dysfunction^123,130-132^. These alterations increase the likelihood of thromboembolic events, such as AMI, stroke, and peripheral arterial disease ^133^. Recent findings emphasize the importance of hepatokines, liver-derived proteins, in facilitating communication between the liver and the heart. Among these, FGF21, fetuin-A and angiopoietin-like proteins (ANGPTLs) play distinct, opposing roles in the development of cardiac dysfunction associated with MASLD^134-136^.

These findings underscore the complex and multifaceted relationship between MASLD and CVD. While MASLD clearly elevates the risk of various CVE, the extent to which it independently influences cardiovascular mortality remains an area requiring further investigation, particularly in relation to disease severity, fibrosis stage, and underlying metabolic dysfunctions such as IR.

### Alcoholic Liver Disease and CVD

Chronic alcohol consumption is a significant risk factor for liver diseases which in turn can profoundly affect cardiovascular health. Alcohol-associated liver disease (ALD), formerly known as alcoholic liver disease, represents a continuum of liver injuries resulting from excessive alcohol consumption, ranging from hepatic steatosis to severe manifestations such as alcohol-associated hepatitis, alcohol-associated cirrhosis, and acute-on-chronic liver failure^137^. Its progression typically begins with steatosis, which may advance to more severe stages depending on factors such as prolonged heavy alcohol consumption, female sex, genetic and epigenetic predisposition, dietary habits, race and ethnicity, and the presence of comorbidities^137^. The recent multisociety Delphi consensus introduced the term metabolic dysfunction-associated ALD (MetALD)^138^ to describe patients with MASLD, who also consume alcohol in excess. MetALD has been linked to a greater hepatic fibrosis burden compared to MASLD, indicating that alcohol consumption may synergistically exacerbate clinical phenotypes and worsen prognoses in individuals with metabolic dysfunction^139^.

A large systematic review including 50,302 individuals with ALD revealed a markedly increased risk of CVD, one of the leading causes of death in this population. In those with alcohol-associated hepatic steatosis, the risk of CVD-related mortality was 40% higher compared to controls, rising to fivefold in individuals with alcohol-associated hepatitis and 2.5-fold in those with cirrhosis^140^. In a Swedish cohort of 3,488 individuals with ALD, the risk of CVD was more than double in the first year following an ALD diagnosis, with a cumulative incidence of 12% at five years versus 6% in the general population, and a modestly elevated risk persisting even after 10 years, before leveling off due to competing liver-related mortality^141^. Interestingly, accumulating evidence have also shown that while both MASLD and ALD are linked to increased cardiovascular risk, ALD is associated with higher overall and CVD-related mortality^142,143^. The study utilizing 7,980 NHANES III participants with SLD, categorized into subgroups (pure MASLD, MetALD, ALD with metabolic dysfunction, and non-MASLD steatosis), demonstrated that patients with MetALD and ALD with metabolic dysfunction had a higher mortality compared to those with pure MASLD or non-MASLD steatosis^144^. In a study of 105,328 Korean individuals, the population was categorized into 34,382 with MASLD, 8,319 with ALD, and 10,098 with excessive alcohol consumption (EAC) but without steatotic liver disease. Both NAFLD and ALD were significantly associated with the presence of coronary artery calcium (CAC), a marker of subclinical atherosclerosis, with ALD showing a slightly stronger association than MASLD, highlighting the added cardiovascular burden of alcohol consumption in the presence of liver steatosis. Alcohol consumption was also linked to an increased risk of CAC, though the association was less pronounced compared to ALD or MASLD, suggesting that even without steatotic liver disease, excessive alcohol intake independently contributes to cardiovascular risk^145^. Another study utilizing a large Korean cohort including 165,654 individuals with MASLD registered a 19% higher risk of CVE compared to the general population, while 22,521 with MetALD and 7,416 with ALD exhibited 28% and 29% higher risks, respectively^146^. These findings highlight the compounding effects of alcohol on the liver and cardiovascular system, as both MetALD and ALD showed elevated risks beyond those observed in MASLD, likely driven by alcohol-induced liver damage, systemic inflammation, and metabolic dysfunction. This risk was evident across obese and non-obese individuals and in different stages of hepatic steatosis and fibrosis, suggesting shared mechanisms such as systemic inflammation and metabolic dysfunction^145^.

Alcohol-related liver damage is primarily attributed to the toxic effects of ethanol and its highly reactive metabolite, acetaldehyde. Alcohol metabolism via alcohol dehydrogenase (ADH) and cytochrome P450 2E1 (CYP2E1) produces acetaldehyde as a reactive intermediate. Chronic ethanol consumption exacerbates acetaldehyde accumulation in the liver by increasing its production and impairing its detoxification through aldehyde dehydrogenase (ALDH) enzymes, with ALDH2 serving as the primary isoform responsible for acetaldehyde clearance in the liver ^147^. Ethanol and acetaldehyde also disrupt hepatic lipid metabolism, rapidly inducing steatosis by promoting lipogenesis and inhibiting fatty acid oxidation as an acute response to alcohol abuse^148^. Additionally, acetaldehyde directly damages cardiac myocytes, resulting in contractile dysfunction^149^.

Acetaldehyde has been shown to stimulate type I collagen synthesis in hepatic stellate cells (HSC) through activation of Protein Kinase C (PKC) and Nuclear Factor kappa-light-chain-enhancer of activated B cells (NF-κB)^150,151^. Chronic alcohol exposure shifts alcohol metabolism predominantly to the CYP2E1 pathway^152,153^, leading to the production of reactive oxygen species (ROS) and hydroxyethyl radicals. These byproducts contribute to oxidative stress and lipid peroxidation, which are key drivers in the pathogenesis of ALD. Oxidative stress induces the release of pro-inflammatory cytokines^154,155^, including tumor necrosis factor-alpha (TNF-α)^156,157^, interleukin-6 (IL-6)^158^, interleukin-1 beta (IL-1β)^159^, and high mobility group box 1 (HMGB1)^160,161^. These cytokines exacerbate liver inflammation and damage while also increasing systemic cardiovascular risk.

ALD is associated with metabolic dysregulation, including IR and dyslipidemia- both established risk factors for CVD. Chronic alcohol intake further alters lipid profiles and promotes visceral fat accumulation, contributing to heightened cardiovascular morbidity^140^.

In conclusion, the cardiovascular consequences of ALD are complex and multifaceted, highlighting the need for a thorough understanding of the underlying mechanisms and contributing risk factors to inform prevention and management strategies.

### Cirrhosis and Cirrhotic Cardiomyopathy

Cirrhosis, the common final stage of severe liver disease, is characterized by a complete derangement of hepatic architecture, promoted by formation of regenerative nodules encased by fibrous bands as a reaction to chronic liver damage, ultimately resulting in portal hypertension, liver failure^162^, and extra-hepatic complications.

Cardiometabolic consequences of cirrhosis due to hepatic insufficiency include impaired glucose metabolism (hyperglycemia due to reduced catabolism of glucagon and growth hormone) and increased IR, followed, in later stages, by hypoglycemia due to decreased insulin catabolism and reduced gluconeogenesis/glycogenolysis^163^, altered protein metabolism (hypoalbuminemia and ascites, loss of muscle proteins)^164^, and lipid metabolism (hypocholesterolemia^165^ with alterations of cell membranes, and lipids malabsorption due to reduced bile acid synthesis^166^.

Due to the continuous liver-heart interplay, a severe complication of cirrhosis is cirrhotic cardiomyopathy (CCM). CCM is a chronic clinical condition observed in patients with cirrhosis (irrespective of the etiology), defined by a combination of reduced myocardial contractile response to stress (physiological, pathological, or pharmacologic stress), diastolic dysfunction, and electrophysiological abnormalities, occurring in the absence of any other known cardiac disease^167^. Approximately 60% of patients with cirrhosis are estimated to have CCM^168^. Arterial vasodilation, central hypovolemia, and hyperdynamic circulation, together with portal hypertension contribute to its development, along with hepatorenal syndrome and hepatopulmonary syndrome^169-171^. Portal hypertension triggers splanchnic vasodilation via liver-derived vasodilators (e.g., NO, carbon monoxide, prostacycline)^172,173^. This reduces systemic vascular resistance and arterial pressure, and redistributes blood flow, leading to central hypovolemia^174^. Portosystemic shunting and bacterial translocation further exacerbate central hypovolemia, activating the sympathetic nervous system and causing a hyperdynamic circulation with increased heart rate and cardiac output^21,175^.

Cirrhotic patients exhibit reduced CO during physical stress compared to non-cirrhotic individuals, primarily due to an insufficient heart rate response and diminished myocardial contractility during exercise^176^. Mediators of impaired contractility include long-term exposure of cardiomyocytes to high levels of noradrenalin usually present in cirrhosis, resulting in internalization, sequestration, and down regulation of β-adrenergic receptors on plasma membrane^177^.

Diastolic dysfunction develops early and characterizes CCM, often preceding systolic dysfunction, with a prevalence in cirrhotic patients from 43% to 70%^178^. Unlike LV systolic failure, which typically occurs under stress, echocardiographic signs of diastolic dysfunction may also be present at rest^179^. The underlying pathophysiology of diastolic dysfunction involves increased myocardial wall stiffness, likely due to a combination of mild myocardial hypertrophy, fibrosis, and subendothelial edema^180^. Preclinical data from cirrhotic rats support the hypothesis that diastolic stiffness is due to altered titin structure, a protein responsible for cardiomyocytes’ stiffness muscle^181^.

QT-interval prolongation, caused by defective K+ channel function in ventricular cardiomyocytes, occurs in 30-60% patient with CCM^182^ and might help identify cirrhotic patients at risk of CCM.

No specific treatments are available for CCM. However, in addition to diuretics, non-selective betablockers can help reduce hyperdynamic load and improve QT interval^179^. Angiotensin-converting enzyme (ACE) inhibitors should be used with caution in patients with CCM, as they may worsen renal function and exacerbate the existing vasodilation associated with advanced cirrhosis^170^.

## 3 CVD driving liver disease: Heart Failure (HF)

The spectrum of CVD affecting liver function largely converges in HF, representing an intermediate or advanced stage occurring in the progression of many cardiac diseases.

HF is a clinical syndrome were structural or functional abnormalities of the heart lead to signs and symptoms of congestion and hypoperfusion^183^. Mechanistically, cardiac dysfunction hinders the heart’s ability to maintain sufficient perfusion of peripheral organs, or an increase in cardiac filling pressures is required to guarantee adequate CO.

HF affects 1–2% of the global population and impose a significant burden of morbidity and mortality^184^.

Several HF groups and phenotypes exists, each characterized by distinct etiologies and pathophysiology. The earliest and best studied form of HF is heart failure with reduced ejection fraction (HFrEF), in which LV ejection fraction (LVEF) – the most commonly adopted indicator of LV systolic function – is ≤40% at rest, thus significantly impaired^185^. Several etiologies may cause HFrEF, with IHD, genetic and acquired cardiomyopathies affecting myocardial contractility being the most common^186^.

All HFrEF etiologies converge in a homogeneous pathophysiology and natural history, involving renal hypoperfusion, SNS and RAAS activation, sodium and fluid retention, release of mediators of adverse cardiac remodeling and progression to arrhythmic events or end-stage hemodynamic failure and cardiovascular death^187^. Some of these mechanisms overlap with those observed in cirrhosis, such as altered SNS response, secondary hyperaldosteronism and fluid retention and shared therapies exists (e.g. mineralocorticoid receptor antagonist – MRA and betablockers)

HFpEF accounts for more than 50% of total HF cases and its prevalence is expected to soon surpass HFrEF^186,188^. HFpEF is a largely heterogeneous syndrome. Among the different HFpEF phenotypes, the cardiometabolic one, is driven by metabolic syndrome. Cardiometabolic HFpEF, is arguably the most common HFpEF phenotype, since approximately 80% of HFpEF patients are overweight or obese^189,190^ and metabolic comorbidities such as T2D and dyslipidemia are highly prevalent in these patients^191-193^.

Cardiometabolic HFpEF is associated with worse quality of life and high cardiovascular mortality^194,195^. Mechanistically, this phenotype is linked to lipid accumulation in the heart (defined as myocardial steatosis^196-199^ and systemic inflammation, making it a form of organ damage related to metabolic dysfunction, similar to MASLD^200^.

In subjects living with obesity and metabolic syndrome, the lipid spill-over from adipose tissue into the bloodstream, promotes lipid accumulation in the heart ^196,197,201^. Lipid accumulation correlates with diastolic dysfunction and cardiac remodeling, including LV hypertrophy and increased left atrial volume^196^. Myocardial triglyceride content also correlates with reduced exercise capacity^198^. Lipid-induced myocardial toxicity and diastolic dysfunction result from different mechanisms including increased fibrosis^202-204^, microvascular dysfunction (resulting in insufficient oxygen supply)^205-207^ and defective mitochondrial function and/or loss of metabolic flexibility, impacting energy metabolism, reducing availability for adenosine triphosphate (ATP)^206,208-212^ for cardiac relaxation and hampering CO increase during exercise^213^, eventually reducing exercise capacity.

Changes in cardiac metabolism are key in HF pathogenesis. In HFrEF, metabolic flexibility is impaired and FAs and glucose oxidation are reduced^214^. Increased oxidation of β-hydroxybutyrate (β-OHB), the primary KB, is observed in HFrEF^215-217^, with elevated circulating β-OHB levels been consistently reported in both animal models and humans, suggesting that HFrEF stimulates endogenous ketogenesis^215,218,219^. Ketogenesis occurs primarily in the liver, which therefore is pivotal to sustain cardiac energetics in these patients. The mechanisms driving increased hepatic ketogenesis in HFrEF remain poorly understood. Reduced cardiac utilization of long-chain FAs may shifts their systemic metabolism toward hepatic β-oxidation, thereby fueling ketogenesis. Additionally, HF is associated with increased natriuretic peptide concentrations, heightened sympathetic tone and elevated catecholamine levels, all of which promote adipose tissue lipolysis and increase circulating FAs^220-222^.

At least in the short term, the shift toward KBs is an adaptive mechanism of the disease^223,224^. Importantly, impaired ketogenesis has been described in MASLD, especially when β-OHB levels are measured after prolonged fasting^225,226^, with lower ketogenic capacity in patients with advanced disease stages^227,228^. Whether the coexistence of impaired ketogenesis in patients with MASLD contribute to worse cardiac energetics in HFrEF is unknown. Importantly, if in HFrEF KBs serve as alternative energy source, KB utilization in HFpEF remains less defined. As observed in different animal models, KB oxidation is unchanged/reduced in HFpEF animals compared with non-failing controls, with reduced expression of β-hydroxybutyrate dehydrogenase 1 (BDH1), a key enzyme for KB oxidation^208-210^. The role of impaired liver ketogenesis in HFrEF vs HFpEF patients remains largerly unexplored.

### Heart-liver hemodynamic interactions in HF: congestive hepatopathy (CH)

One of the most common mechanisms by which HF affects liver function is congestive hepatopathy (CH), a common complication seen in approximately 20–30% of chronic HF cases^229^. CH, also known as cardiac hepatopathy, primarily results from elevated central venous pressure (CVP), a hallmark of right heart failure (RHF). CVP increase causes chronic hepatic congestion, potentially progressing to fibrosis, cirrhosis, or HCC. Although most frequently linked with RHF, CH can also occur in conditions such as constrictive pericarditis and severe tricuspid regurgitation.

CH develops due to disrupted hepatic blood outflow. The blood supply reaching the liver from the hepatic artery and portal vein converge in hepatic sinusoids and drain through hepatic veins into the inferior vena cava to return to the right heart^230^. Because hepatic veins lack valves, elevations in right atrial pressure are directly transmitted into the liver, causing passive hepatic congestion^231^. This impairs portal venous inflow and reduces overall hepatic perfusion^232^.

Although the hepatic artery buffer response can compensate for reduced portal flow by increasing arterial inflow^233^, it does not prevent congestion-related injury. Chronic congestion leads to sinusoidal dilation and accumulation of fluid in the perisinusoidal space, compressing adjacent hepatocytes and impairing metabolic function. Oxygen diffusion is also impaired, promoting fibrogenesis^232^. This process underlies the distinctive “nutmeg liver” appearance seen in CH, characterized by mottled red and pale areas that reflect congested and ischemic regions^232^. Prolonged congestion may also compress bile canaliculi and ductulus, impairing bile acid secretion and contributing to cholestasis and impaired drug metabolism^234^.

Elevated sinusoidal pressure also induces shear stress, activates hepatic stellate cells, and decreases NO production by endothelial cells, further accelerating fibrosis^235,236^. Systemic inflammation, oxidative stress, and neurohormonal activation in HF further exacerbate liver dysfunction, establishing a vicious cycle of organ interdependence.

RHF plays a central role in both HF phenotypes, albeit via distinct mechanisms, and its development marks disease progression. In HFpEF, diastolic dysfunction of the LV increases left atrial pressure, which is transmitted backward into the pulmonary circulation, causing pulmonary hypertension^237^. This elevated afterload leads to RV dysfunction—observed in 20–35% of HFpEF patients^238^—leading to RHF and CH. In HFpEF, CH primarily results from sustained venous congestion, although liver perfusion is generally preserved. In HFpEF, CH often coexists with MAFLD further exacerbating liver damage.

In HFrEF, impaired LV systolic function and reduced CO result in elevated left-sided filling pressures. These changes cause pulmonary congestion and secondary pulmonary hypertension^239^. The RV compensates for increased afterload but may eventually fail, resulting in RHF—a key prognostic factor linked with poor outcomes^240^. HFrEF may specifically lead to ischemic hepatitis, when acute drops in CO reduce hepatic artery blood flow, eventually leading to acute liver hypoperfusion.

Many individuals with CH are asymptomatic, with hepatic symptoms often overshadowed by HF. When present, symptoms include right upper quadrant discomfort from hepatomegaly, jaundice due to impaired bilirubin clearance, ascites from elevated venous pressure, nausea, vomiting, and peripheral oedema—though oedema often reflects HF more than hepatic dysfunction^241^. Treatment focuses on optimizing cardiac function and relieving congestion. Diuretics, especially loop diuretics, are key but require careful dosing to avoid hepatorenal syndrome^233^. Sodium restriction and fluid management support therapy, and paracentesis may be needed for refractory ascites^242,243^.

Tricuspid valve disease (TVD), particularly tricuspid regurgitation (TR), can lead to liver damage even in the absence of overt HF. Severe TR can cause retrograde blood flow, leading to elevated CVP. This pressure is transmitted to the hepatic veins, resulting in hepatic congestion. Over time, this congestion can cause hepatocellular injury and fibrosis. In a retrospective study involving 435 patients with severe TR, 14.5% had documented liver disease, and elevated liver enzymes were common^244^. In another cohort, 11% of patients with severe isolated TR exhibited liver disease, highlighting the relevance of hepatic involvement in this population^245^. Importantly, liver stiffness assessed via transient elastography is an independent predictor of adverse outcomes in patients with TR^246^. These findings underscore the importance of assessing liver function in patients with severe TR, even in the absence of HF, as hepatic involvement may influence management strategies and outcomes.

### Acute Heart Failure: Short-term liver damage during heart failure exacerbation

Acute liver hypoperfusion, a condition also known as ischemic hepatitis or acute cardiogenic liver injury (ACLI) mainly occurs in the context of acute HF (AHF), a condition marked by rapid deterioration of signs and symptoms of HF. Several AHF phenotypes exists, spanning from progressive fluid retention slowly turning into symptomatic congestion (as in acute decompensated heart failure - ADHF) to cardiogenic shock, the deadliest from of AHF^247^. Hepatic dysfunction occurs in 20 to 30% of AHF patients^229^.

ACLI arises from a sudden reduction in blood flow and oxygen supply to the liver. This condition is typically associated with acute reductions of CO, where systemic hypoperfusion and ischemia impair hepatocyte oxygenation and metabolism^234^. Clinical manifestations include acute liver injury with elevated aminotransferase levels and acute liver failure in more severe cases ^248^. Histologically, ACLI is defined by necrosis of pericentral zone 3 hepatocytes. Zone 3 hepatocytes, closest to the central vein, are particularly vulnerable due to their reduced oxygen supply compared to hepatocytes in periportal zones 1 and 2, predisposing them to hypoxic damage^249^. This process is often worsened by pre-existing hepatic congestion caused by elevated hepatic venous pressure^249,250^. In patients with chronic CH, even mild reductions in CO may cause ACLI^229^ due to impaired compensatory mechanisms. ACLI is diagnosed when aminotransferase levels rise >20 times over the upper normal limit in the setting of cardiac, circulatory, or pulmonary failure, and other causes of liver damage are excluded^251^. Management primarily targets the underlying AHF^252^. Although some patients recover from the acute phase, with aminotransferase levels normalizing within 3 to 7 days, mortality rates remain high^251^.

### Cardiac Amyloidosis and Hepatic Involvement

Cardiac amyloidosis, particularly transthyretin (ATTR) amyloidosis, is characterized by the deposition of amyloid fibrils in the myocardium, leading to restrictive cardiomyopathy^253^. This condition can also affect the liver, either directly through amyloid deposition or indirectly via cardiac dysfunction. Liver involvement is evidenced by elevated liver stiffness, which correlate with the severity of cardiac amyloidosis. In a cohort of patients with wild-type ATTR cardiac amyloidosis, higher liver stiffness was associated with advanced disease stages and higher mortality^254^. Furthermore, elevated liver enzymes, particularly alkaline phosphatase and transaminases, have been observed in patients with cardiac amyloidosis, indicating hepatic dysfunction^255^. Altered transient elastography due to either CH or deposition of amyloid has been observed also in patients with light chain amyloidosis^256^, suggesting that increased liver stiffness may have several explanations in these patients. These findings highlight the importance of monitoring liver function in patients with cardiac amyloidosis, as hepatic involvement can have prognostic implications and may influence therapeutic strategies.

## 4 Novel Mediators of Bidirectional Crosstalk Between Liver and Heart

### Liver to Heart communication

Hepatokines, liver-derived secreted proteins, are critical mediators linking hepatic metabolism to whole-body homeostasis, particularly in cardiometabolic diseases. Among hepatokines, Coagulation factor XI (FXI), FGF21, and serum amyloid A proteins (SAA1/4) have garnered attention for their roles in cardiac metabolism and remodeling.

FXI has been traditionally known as a coagulation factor, but recently has been implicated in cardiovascular pathology. Elevated FXI levels are associated with thrombo-inflammation, driving endothelial dysfunction and cardiac fibrosis^257-259^. Pro-thrombotic states exacerbate CVD by promoting microvascular injury, platelet activation, and chronic inflammation. Excess FXI activity can also amplify coagulation cascade crosstalk with pro-fibrotic pathways, worsening cardiac remodeling seen in HF. Interestingly, FXI may play a context-dependent cardioprotective role under specific conditions^260,261^. For example, in a preclinical model of HFpEF, FXI overexpression during disease progression surprisingly demonstrated cardioprotective effects. This finding highlights the nuanced role of FXI, where modest levels may stabilize microvascular integrity and endothelial repair, mitigating inflammation and fibrosis. Understanding the threshold at which FXI transitions from protective to pathological remains a key research focus.

FGF21 is a well-established hepatokine that improves metabolic homeostasis under nutrient stress^262-264^. It functions as a systemic mediator of energy balance, enhancing glucose uptake, FA oxidation, and mitochondrial function in peripheral tissues, including the heart^265-267^. Elevated FGF21 levels are observed during metabolic stress, serving as a compensatory response to obesity, IR, and lipotoxicity. In cardiometabolic diseases, FGF21 has potent cardioprotective effects by alleviating lipotoxicity injury and improving myocardial energy efficiency^268,269^. FGF21 reduces cardiac hypertrophy and fibrosis by activating AMPK and PGC1a signaling, which enhances mitochondrial biogenesis and reduces oxidative stress.

Serum amyloid proteins (SAA1/4) are acute-phase hepatokines primarily induced under inflammatory states^270,271^. While essential for host defense, chronically elevated SAA1/4 levels in metabolic syndrome can exacerbate cardiac pathology. SAAs promote systemic inflammation, activate immune cells, and induce extracellular matrix (ECM) remodeling, contributing to cardiac hypertrophy and fibrosis^272-274^. In the heart, SAA1/4 abundance associates with an enhanced pro-inflammatory state and fibrotic gene programs^272,274,275^, likely contributing to maladaptive cardiac remodeling, although the direct evidence of these heart or resident cell-specific effects is lacking. Elevated SAA1/4 levels in obese or insulin-resistance individuals reflect a persistent hepatic inflammatory state that perpetuates cardiovascular dysfunction^270,274,276^.

Hepatokines such as FXI, FGF21, and SAA1/4 exemplify the liver’s critical role in regulating cardiac remodeling and metabolism. FXI’s dual nature underscores the need for context-dependent therapies to modulate its levels safely. Meanwhile, FGF21 emerges as a protective hepatokine, linking metabolic state to cardiomyocyte health, and SAA1/4 highlights the detrimental impact of chronic inflammation on cardiovascular health. Targeting these hepatokines, particularly through interventions via behavioral and/or pharmacological modulation, holds promise for treating the liver-heart axis in cardiometabolic syndrome and preventing CVD progression.

In addition to hepatokines, liver-derived extracellular vesicles (EVs) are increasingly recognized as mediators of hepatic metabolic dysfunction in cardiovascular disease (CVD). MASLD leads to an increased release of EVs enriched in proinflammatory factors (PMID: 25470250), which may propagate systemic inflammatory signalling and reinforce the chronic low-grade inflammation underlying both ASCVD and HF. Steatotic hepatocyte-derived EVs also promote endothelial dysfunction by increasing coronary microvascular permeability and inflammation (PMID: 34838052, PMID: 31568800). Additionally, liver-secreted EVs contribute to the development of metabolic cardiomyopathy by impairing cardiomyocyte mitochondrial function (PMID: 31434696). While the study of liver-derived EVs in CVD is promising, this area remains relatively novel, and further research is needed to establish clear connections and develop therapeutic strategies.

### Heart to liver communication

Growing evidence supports the idea that the heart may influence systemic metabolism, including hepatic metabolism, by functioning as an endocrine organ. A key example of heart-secreted proteins involved in regulating whole-body energetics are the natriuretic peptides, atrial natriuretic peptide (ANP) and brain natriuretic peptide (BNP). While natriuretic peptides are best known for their role in adipose tissue, where they promote lipolysis and increase energy expenditure^277-279^, their receptor, NPR-A, is also expressed in the liver^280^. Infusion of ANP in healthy individuals increases circulating levels of FAs and of β-OHB^281^. Accordingly, a covariant structure analysis identified a positive correlation among plasma levels of KB and BNP in cohort of patients with cardiovascular disorders^282^. In a model of diet-induced obesity, ANP administration significantly reduced hepatomegaly and hepatic steatosis, suggesting a direct role for natriuretic peptides in regulating liver metabolism. ANP has also been shown to modulate hepatic carbohydrate metabolism by inhibiting pyruvate kinase activity^283^.

Beyond natriuretic peptides, cardiac cells secrete a variety of proteins and peptides, collectively referred to as cardiokines. For instance, cardiac fibroblasts and cardiomyocytes secrete immunomodulatory signals in pathological conditions^284,285^. Although the link between myocardial inflammation and liver dysfunction remains unclear, a recent study demonstrated that myocardial ischemia and AMI in particular exacerbates liver damage in MAFLD through two mechanisms: (1) increasing inflammatory monocyte levels and their recruitment to the liver, and (2) secreting periostin (POSTN), an extracellular matrix protein re-expressed by cardiac fibroblasts during cardiac injury^286^. AMIs lead to elevated hepatic triglyceride levels, worsened steatosis, and aggravated fibrosis^287^. POSTN delivery to primary hepatocytes increased lipid accumulation by activating JNK1/2 signaling and reducing peroxisome proliferator-activated receptor α (PPARα) levels^287^. Another heart-liver axis in AMI involves interleukin-6 (IL-6) and hepatic STAT3 signaling. STAT3 activation suppresses the mineralocorticoid receptor and upregulates hepatic FGF21^288^. Given the role of IL-6 in liver homeostasis, including regeneration, insulin signaling, and glucose metabolism^289^, AMI-induced IL-6 release may contribute to additional hepatic alterations. Indeed, transcriptional analysis of metabolically active tissues during AMI progression revealed dysregulation of immune response and fatty acid metabolism pathways in the liver^290^.

Follistatin-like 1 (FSTL1), a glycoprotein secreted by cardiomyocytes and fibroblasts in response to pathological stimuli such as pressure overload and ischemia/reperfusion, also plays a role in systemic metabolism^291^. Elevated circulating FSTL1 levels are associated with poor prognosis in AMI^292-294^. FSTL1 exerts cardioprotective effects by enhancing cardiomyocyte survival, reducing apoptosis, and modulating inflammation and energetics^291,295,296^. Beyond its local actions, FSTL1 influences systemic metabolism by increasing FA oxidation and altering circulating levels of FAs, glucose, and KBs. Treatment of cardiomyocytes and myotubes with FSTL1 enhances AMPK phosphorylation and mitochondrial respiration^296^. While its hepatic effects remain unclear, muscle-derived FSTL1 has been implicated in MASH pathogenesis via a skeletal muscle-liver axis, and recombinant FSTL1 increases triglyceride accumulation in primary hepatocytes exposed to palmitic acid^297^.

Another example of heart-liver interaction involves secreted phospholipase A2 (sPLA2), an enzyme highly expressed in the heart that hydrolyzes phospholipids to release FAs and lysophospholipids. sPLA2 expression increases after AMI^298^, and its secretion by cardiomyocytes, mediated by the chemokine MCP-3, leads to hepatic prostaglandin E2 release. This dysregulates liver X receptor α (LXRα) and sterol regulatory element-binding protein 2 (SREBP-2) signaling, altering inflammatory and lipid metabolic gene expression and increasing hepatic triglycerides and VLDL levels^299^.

Growth differentiation factor-15 (GDF15), a member of the transforming growth factor-β (TGF-β) superfamily, is another cardiokine with systemic effects. While not expressed in the healthy adult heart, GDF15 is secreted by cardiomyocytes under stress conditions such as ischemia/reperfusion and nitrosative stress^300^. Elevated GDF15 levels are associated with poor outcomes in HF^301-303^. GDF15 is also secreted in other conditions, including exercise, obesity, and aging^304-306^, and acts centrally to suppress appetite and regulate energy balance^307,308^. In pediatric heart disease, cardiomyocyte-derived GDF15 impairs growth hormone signaling in the liver, contributing to growth failure^309^. Recently, GDF15 was shown to improve insulin sensitivity in diet-induced obesity by enhancing β-adrenergic signaling in tissues including the liver^310^. Further research is needed to clarify its role in heart disease.

Finally, Mitsugumin 53 (MG53), an E3 ligase secreted by cardiomyocytes and myocytes in response to elevated glucose or insulin, modulates whole-body insulin sensitivity. MG53 inhibits insulin receptor signaling via extracellular blockade and intracellular ubiquitination, reducing Akt phosphorylation in the liver, skeletal muscle, heart, and visceral fat^311^.

While evidence supports the heart’s ability to signal to the liver via endocrine factors, only a few heart-secreted factors have been characterized to date. For many of these, their impact on liver physiology and mechanisms of action, particularly in cardiac disease, remain poorly understood. Further research is needed to fully elucidate these heart-liver interactions and their implications for systemic metabolism.

## 5 Methods to Study Heart-Liver Interactions

### Preclinical models of liver and heart disease

Animal models of liver and heart disease remain essential for the discovery of novel mechanisms of liver-heart interactions and to explore mechanistic implications of targets leveraged from clinical cohorts. Navigating through the many animal models of liver and heart disease can be challenging. Here, we described animal models recapitulating key features of liver (MASLD, ALD, cirrhosis) and heart (AMI, HFrEF, HFpEF) diseases, highlighting which may be suitable to answer specific research questions along the liver-heart axis.

### MASLD

Several animal models of MASDL exists, generated through diets, genetic manipulation or administration of different chemicals. Each model is different in terms of metabolic traits (obesity, IR, dyslipidemia), liver damage, and histopathological features such as steatosis, ballooning, inflammation, and fibrosis. As clarified in dedicated comparisons of MASLD animal models ^312,313^, high-fat, high-fructose (HFHS) diets models are the most accurate in recapitulating MASLD hallmarks observed in humans. Examples are the DIAMOND model (C57BL/6J mice fed with high-fat, high-carbohydrate diet)^314^ and the AMLN diet model (HFHS diet)^315^, both presenting progressive steatosis, lobular inflammation, hepatocyte ballooning, and fibrosis, together with IR and dyslipidemia. In genetic models, such as the hyperphagic MC4R-knockout mice exposed to a Western diet, the combination of high-fat diet (HFD) and genetic manipulation accelerates the progression of liver disease (including advanced fibrosis), systemic metabolic dysfunction and development of HCC^316^. The abovementioned models can be used to simultaneously explore the contribution of dietary and genetic metabolic stressors to the liver and the heart, exploring also metabolic interorgan signaling.

To specifically investigates the role of liver disease in driving cardiac changes, in the absence of obesity, specific models can be used. The STAM model combines neonatal streptozotocin injection with high, leading to diabetes, steatohepatitis, and eventually fibrosis and HCC. It lacks features like obesity and dyslipidemia, specifically excluding this pathogenetic aspect from the phenotype development^317^. Similarly, the methionine- and choline-deficient (MCD) diet induces rapid liver injury and fibrosis but lacks systemic metabolic features such as obesity or insulin resistance^318^.

### ALD

To date, no single animal model fully replicates the complexity of human ALD, but several models (comprehensively reviewed in dedicated articles^319^) can be adopted to answer specific research questions. Importantly, ALD animal models can be used to explore the cardiac implication of alcohol abuse. The Alcohol in Drinking Water (ADW) models, are simple models inducing steatosis and mild inflammation over weeks^320,321^. However, relaying on voluntary intake, limits blood alcohol concentrations (BAC) and disease severity. The Drinking in the Dark (DID) model enables higher BAC and mimics binge patterns but is mostly limited to early liver damage^322,323^. The Lieber-DeCarli (LdC) diet^324,325^ enhances ethanol intake by incorporating alcohol into a liquid diet, leading to controlled, chronic exposure with reliable steatosis and inflammation in 4–12 weeks. It is ideal for studying early ALD but lacks fibrosis unless combined with a second hit (e.g., carbon tetrachloride - CCl_4_ or lipopolysaccharide - LPS)^326-328^. A powerful variation is the NIAAA model^329^, which adds an acute ethanol binge to the LdC protocol, producing more severe steatohepatitis and immune infiltration. The Tsukamoto-French model^330,331^, using intragastric ethanol infusion, remains the most comprehensive, achieving high sustained BAC and advanced histological features such as necrosis, fibrosis, and inflammation, closely resembling human ALD. Despite its technical complexity, it is the most robust model for replicating progressive liver injury.

Importantly, despite in both rodents and humans, chronic ethanol consumption is expected to induce dilated cardiomyopathy^332,333^, little is known about cardiac function and/or HF in most of ALD animal models.

### Animal models of advanced chronic liver disease

Among the most widely utilized preclinical models of advanced chronic liver disease are CCl_4_, thioacetamide (TAA), and common bile duct ligation (cBDL), each offering distinct advantages and limitations in terms of translational relevance and feasibility. The CCl_4_ model is historically considered the reference standard for toxicant-induced advanced chronic liver disease^334,335^. It closely replicates critical histopathological features of human cirrhosis, including hepatocellular necrosis, hepatic stellate cell activation, sinusoidal capillarization, and regenerative nodule formation. Chronic administration, particularly via intraperitoneal injection^336^ or inhalation^337^, can lead to decompensated cirrhosis characterized by ascites and portal hypertension. However, this model is highly dependent on animal strain, with BALB/c mice and Wistar rats showing greater susceptibility^338^. Additionally, variability in fibrosis severity and partial reversibility upon cessation of the toxicant are notable limitations. The TAA model, while less commonly used, is increasingly recognized for its high reproducibility and greater histological similarity to human cirrhosis^339,340^. TAA-induced fibrosis exhibits a stable periportal and lobular distribution with prominent regenerative nodules, persisting even after toxicant withdrawal^341^. Administration is feasible via intraperitoneal injection or drinking water^342^, with low mortality and minimal technical complexity. Unlike CCl_4_, TAA-induced cirrhosis is less influenced by genetic background, making it suitable for broader applications^343^. The cBDL model is a well-established surgical model for secondary biliary cirrhosis, inducing fibrosis through biliary obstruction and cholestasis^344,345^. It yields rapid, consistent cirrhosis in 3–4 weeks and is particularly relevant for studying cholestatic liver injury^346^. Nevertheless, it requires surgical expertise and carries a moderate mortality risk, primarily due to bile duct complications. Moreover, its utility in pharmacological studies is limited due to impaired biliary excretion. Overall, TAA emerges as the most reproducible and histologically relevant model of human advanced chronic liver disease, while CCl_4_ remains preferable for studies involving decompensation and portal hypertension. Despite CCM is a well-known organ damage occurring in cirrhotic patients, cardiac disease in animal models of advanced liver disease is largely unexplored.

### HFrEF and IHD animal models

In preclinical research on HFrEF, small animal models—particularly rodents—are often indispensable due to their affordability, ease of handling, and suitability for high-throughput and mechanistic studies. A detailed description of HFrEF animal models can be found in dedicated reviews^347^, a brief overview of most suitable models will follow.

Pressure overload models, such as transverse aortic constriction (TAC), replicate the pathophysiological progression from concentric hypertrophy to HFrEF^348^. Though technically demanding, advancements in TAC procedures have enhanced reproducibility and reduced mortality^349-352^. Permanent left anterior descending coronary artery ligation is the most widely used method to induce AMI and is the most adopted model of ischemic HFrEF^353^. Drug-induced models, such as with doxorubicin^354,355^, isoproterenol^356-358^, or angiotensin II^359^, are non-surgical and induce cardiomyocyte injury, fibrosis, and reduced LVEF. These are particularly relevant for studying chemotherapy-related or hypertensive heart disease. Despite less technically demanding, translation may be limited (in the case of doxorubicin models) and most are strain-sensitive, emphasizing the importance of model selection^359^.

Despite anatomical and physiological differences—such as faster heart rates and smaller heart size—rodents remain highly valuable due to their experimental tractability and availability of genetic tools. In comparison, large animal models (e.g. swine models of ischemic HFrEF^360^, or aortic constriction and valvular injury models^361-363^) offer closer physiological and anatomical parallels to humans but are resource-intensive and logistically challenging.

Overall, rodent models—particularly TAC and AMI—provide the best balance of feasibility, reproducibility, and mechanistic relevance, making them the cornerstone of HFrEF research. Little is known about liver damage (e.g. congestion, altered transaminases levels, fibrosis) occurring in HFrEF animal models and if therapeutic interventions aiming at cardiac improvements also affects the liver.

### HFpEF Animal Models

Several HFpEF animal models exists^364,365^. For a model to be reliable, it must replicate clinical signs and symptoms of HF, such as lung congestion (reflected as increased lung weight in mice) and dyspnea (modeled as reduced exercise capacity). Importantly, isolated diastolic dysfunction with preserved LVEF reflects a preclinical state, not HF. It’s essential to define the specific HFpEF phenotype an animal model represents. Since HFpEF can arise from diverse causes like obesity, aging, or hypertension, appropriate models should match the phenotype of interest. Each HFpEF phenotype may differentially impact on liver function and metabolic vs non-metabolic liver disease may specifically lead to different HFpEF subtypes.

The HFD + L-NAME model is a widely adopted cardiometabolic HFpEF murine model^206^. Mice fed a HFD and exposed to NO synthase inhibition via L-NAME develop obesity, hypertension, endothelial dysfunction, insulin resistance, and diastolic dysfunction, all while preserving LVEF. Hepatic steatosis and mild inflammation are also observed, providing a valuable tool for studying MASLD–HFpEF interactions^206^.

The ZSF1 obese rat, a cross of Zucker diabetic fatty and spontaneously hypertensive rats, exhibits hypertension, hyperinsulinemia, hyperlipidemia, renal impairment, and cardiac diastolic dysfunction. While hepatic involvement is less pronounced, with mild steatosis and early fibrosis, this model closely resembles the systemic metabolic syndrome seen in patients^366^. Older C57BL/6J mice fed a Western or HFD in combination with angiotensin II infusion can also develop HFpEF-like features over time. These include diastolic dysfunction, inflammation, and myocardial hypertrophy^367^. However, little is known about hepatic pathology in aging HFpEF models. Hypertensive models using DOCA-salt, or aldosterone infusion induce diastolic dysfunction^368-370^ but lack obesity or IR, focusing on non-metabolic HFpEF phenotypes. Recently, a novel two-hit mouse model of HFpEF has been developed, in which hypertension, one of the principal drivers of HFpEF, is induced via adenoviral-mediated renin overexpression, in combination with HFD. This model exhibits the classical features of HFpEF, including obesity, insulin resistance, hypertension, diastolic dysfunction, and reduced exercise capacity (PMID: 39747575). Although this model remains supra-physiological, it has the advantage of inducing hypertension through activation of the RAAS, a pathway frequently upregulated in cardiometabolic HFpEF (PMID: 31926856).

TAC models may also be employed to study HFpEF, although they require milder constriction than that used in HFrEF models to preserve ejection fraction (PMID: 33969009). When combined with a HFD, this approach recapitulates key haemodynamic features observed in HFpEF patients. As such, it represents a valuable complement to the established HFD + L-NAME model, with the potential for cross-validation between preclinical models improving translational relevance.

In conclusion, despite several animal models of liver and heart disease have been developed over the years, interorgan involvement have been poorly addressed. Similarly, the effects of specific interventions have been invariably assessed on one organ, leaving the other unexplored. Choosing the best strategy to model the organ damage of interest, and testing multiple hits (i.e. combining models) may help in gathering mechanistic data on liver-heart interorgan crosstalk.

### Systems Biology Approaches: Integration of omics to map heart-liver communication

Systems biology approaches are novel research tools for uncovering complex inter-organ signaling pathways by integrating large-scale multi-omics datasets. One of the most powerful techniques in this area is the Quantitative Endocrine Network Interaction Estimation (QENIE) method^371,372^. The core premise of QENIE is that if a signaling relationship exists, the expression of a gene encoding a secreted protein in the origin tissue will correlate with expression changes in genes of the target tissue. QENIE leverages gene expression data from genetically diverse populations, such as the Hybrid Mouse Diversity Panel (HMDP), or human cohorts (i.e. GTEx), to identify secreted proteins in the origin tissue (e.g., liver) that correlate with gene expression patterns in target (e.g., heart). Biweight midcorrelation (bicor)^373^ is used to assess cross-tissue associations, and the calculated Ssec score ranks secreted proteins based on the strength of their correlations with genes in target tissues. Integration with secretome databases (e.g., UniProt) and tissue-specific expression profiles further refines candidate prioritization.

A QENIE framework has been successfully used to identify three liver-derived secreted proteins, namely HGFAC, C8G, and FXI, with potential roles in liver-heart crosstalk^260^. Through cross-tissue transcriptomic correlation in the HMDP, FXI emerged as one of the most interesting targets in liver-heart crosstalk^260^ and was validated as a cardioprotective factor, able to improve diastolic function and attenuate cardiac inflammation and fibrosis. FXI’s proteolytic activity was shown to be essential for cleaving and activating BMP7, leading to the suppression of inflammation- and fibrosis-related gene expression^260^. HGFAC (hepatocyte growth factor activator) was identified as an additional cross-talk candidate ^260^ and its specific role in driving HFpEF phenotype is under investigation. The third candidate, C8G (complement component C8 gamma chain), was shown to reduce heart weight in the HFpEF model^260^. Recently, applying a multi-tissue transcriptomic approach in a mouse model of HFpEF, among 86 liver-secreted candidates, serum amyloid A proteins (SAA1 and SAA4) emerged as HFpEF-specific mediators of liver-heart crosstalk^374^. Circulating levels of these proteins were increased in HFpEF mouse models and in human HFpEF and MASLD cohorts. Notably, their expression was correlated with cardiac fibrosis and extracellular matrix remodeling pathways. Taken together, these studies underscore the critical role of liver-to-heart signaling in driving key features of HFpEF and showcase the power of integrated -omics and systems genetics to reveal novel mediators of liver-heart communication and beyond.

Mediators of interorgan crosstalk can be also investigated using web-based informatic hypothesis-generating interfaces, such as the HMDP systems genetics webpage (https://systems.genetics.ucla.edu/HMDP/) or the Gene-derived correlation across tissues (GD-CAT) webpage (https://pipeline.biochem.uci.edu/gtex/). The former allows to identify statistical associations between genes, SNPs, and/or clinical traits of interest through a range of datasets, such as, mice fed a standard chow-diet, a high-fat high-sucrose (HFHS) diet, and mice exposed to isoproterenol to induce HFrEF. This tool allows to search and generate Manhattan plots, look up quantitative trait loci (QTL), identify gene, SNP, and trait correlations, and visualize genome-wide hotspots; all in a manner that allows the user to generate new and interesting investigatory directions. Utilizing much of the same data in a uniquely distinct manner, the latter (GD-CAT), offers a more focused view of cross-tissue communication built from QENIE methods. GD-CAT allows one to peer into HMDP data (chow diet or HFHS diet fed mice) and GTEx (male, female, or combined) data to investigate cross-tissue gene expression associations. By serving as an easy to use, efficient, *in silico* hypothesis generating and/or solidifying system, GD-CAT is an extremely valuable tool to explore novel secreted factors mediating liver-heart interactions.

Approaches like QENIE, cross-tissue transcriptomic correlation, and proteomic validation enable a comprehensive understanding of the endocrine networks involved in cardiometabolic disease. Web-based informatic interfaces like the HMDP Shiney app and GD-CAT make novel or previously underappreciated cross-tissue connections easy-to-use and thus accessible to the whole scientific community.

### Secretome Profiling: Advances in proximity labeling techniques to track inter-organ signaling molecules

Proximity labeling has emerged as a cutting-edge technique for tagging proteins synthesized in specific subcellular compartments within living organisms. This method relies on engineered biotin ligase enzymes that, in the presence of a substrate, covalently tag endogenous proteins within a radius of a few nanometers. The biotinylated proteins can then be isolated using streptavidin-based enrichment and identified via mass spectrometry^375^. Recent advancements in biotin ligase engineering, enabling faster and more efficient labeling *in vivo*, have expanded the application of this technique to in vivo secretome studies^376^.

Proximity labeling is particularly valuable for studying interorgan communication in both physiological and pathological conditions for several reasons. First, the biotin ligase enzyme can be expressed in a cell-type-specific manner using adenoviral-associated vectors (AAVs) driven by tissue-specific promoters^377,378^ or through transgenic animal models crossed with Cre recombinase lines^379,380^. This allows researchers to precisely determine the cellular and tissue origins of secreted proteins in the animal model of interest (i.e. mouse models of MASDL and/or HFpEF) Second, the biotinylation event is initiated only upon substrate delivery, providing temporal control over the labeling process. This flexibility enables researchers to tailor the labeling period to the specific context, whether it involves acute events (hours) or chronic conditions (days).

To date, cell-type-specific proximity labeling has been successfully used to profile the liver secretome under various physiological and disease conditions. For example, using the TurboID system, a recent study identified carboxylesterase enzymes CES2A and CES2C as the most exercise-responsive proteins secreted by the liver. Further characterization revealed their anti-obesogenic and anti-diabetic properties, uncovering new mechanisms of tissue-tissue communication underlying the benefits of physical activity^381^. Another study demonstrated that hepatic secretion of the glycoprotein Fetuin-A is altered in insulin-resistant conditions^378^. While elevated circulating Fetuin-A levels are associated with MASLD and IR^382^, they are reduced in HF patients with liver hypoperfusion, suggesting impaired hepatic secretion in these patients^383^.

Importantly, proximity labeling can also capture proteins secreted via non-conventional pathways, which may play a critical role in certain conditions. For instance, targeting the biotin ligase to the endoplasmic reticulum (ER) allows for the identification of proteins secreted through the conventional ER-to-Golgi pathway, while targeting the cytoplasmic compartment enables the detection of proteins undergoing unconventional export. Using this dual approach, Wei W. et al. revealed that a HFHS diet suppressed conventional protein secretion while strongly inducing the unconventional secretion of the enzyme betaine-homocysteine methyltransferase (BHMT). This highlights the importance of studying unconventional secretion pathways in metabolic diseases^377^.

Given its versatility, proximity labeling is a powerful tool for investigating cell-specific secretomes and inter-organ communication mechanisms in cardiometabolic diseases, including HFpEF. By providing spatial, temporal, and cell-type-specific resolution, this approach holds great promise for uncovering novel biomarkers and therapeutic targets in complex diseases.

### Modeling inter-organ cross-talk: Organoids, Organ-on-Chip, and Precision-cut-tissue slices

To understand disease mechanisms and develop effective treatments, researchers require models that accurately mimic the complexities of human biology. Traditional *in vitro* models like 2D cell cultures lack the structural and functional complexity of human tissues, while animal models, though valuable, are expensive, time-consuming, and may not fully replicate human biology due to genetic and physiological differences ^384,385^. The use of patient-derived material represents a highly relevant and versatile model for bridging the gap between pre-clinical models and translational research. Such material can be used for systems such as organoids, organ-on-chip and precision-cut tissue slices. Importantly, these systems can be used for the generation of -omic datasets (transcriptomics, proteomics, metabolomics), correlation of data with clinical features, and can serve as a platform for secretome studies.

### Organoids

Recent advancements in organoid and organ-on-chip systems have transformed *in vitro* modeling by providing physiologically relevant platforms for studying organ development, disease mechanisms, and therapeutic responses. Organoids are three-dimensional, self-organizing structures derived from human cells ^386,387^ that closely mimic the architecture and functionality of native tissues. Organ-on-chip systems integrate microfluidic technologies that replicate organ-level functions by using hollow channels lined with living cells under controlled fluid flow. These systems can be interconnected to create multi-organ platforms, enabling the study of complex physiological processes and systemic interactions ^388^. Together these models bridge the gap between traditional *in vitro* and *in vivo* models, enabling the investigation of cell-cell and inter-organ interactions, such as those between the heart and liver. This represents a significant leap forward in our ability to study complex biological systems and address human diseases with unprecedented precision.

Hepatic organoids, derived from sources such as primary liver tissue, embryonic stem cells (ESCs), or induced pluripotent stem cells (iPSCs), have emerged as valuable *in vitro* models for hepatology research^389^. They recapitulate key hepatic functions such as metabolism, detoxification, and bile acid synthesis, making them extremely valuable for studying liver disorders and metabolic diseases. For instance, an ALD model co-culturing hepatocyte organoid with mesenchymal cells has been shown to replicate key phenotypes associated with ALD, such as oxidative stress, steatosis, and fibrosis upon alcohol exposure^390^. Similarly, multicellular organoids with hepatocytes, stellate, and Kupffer-like cells were shown to recapitulate features of MASLD, including steatosis and inflammation^391,392^. Additionally, organoids derived from patients with MASH have been shown to preserve MASH phenotypes, including decrease in albumin production, steatosis, and sensitivity to apoptosis^393^, providing powerful tools for investigating liver pathophysiology, disease progression, and testing therapeutic candidates.

Cardiac organoids, particularly engineered human myocardium (EHM) derived from human-induced pluripotent stem cell-derived cardiomyocytes (hiPSC-CMs), have emerged as a valuable tool for modeling cardiac diseases such as HF. These systems replicate pathological features like contractile dysfunction, hypertrophy, and cell death under chronic stress conditions, such as catecholamine exposure^394^. Advances in cardiac organoid development are extending their utility to broader cardiovascular research, including arrhythmias and myocardial infarction.

### Organ-on-chip

Organ-on-chip technology bridges the gap between static *in vitro* models and dynamic physiological conditions by incorporating microfluidic systems to simulate blood flow and nutrient exchange^395^. These systems enable co-culture of liver and heart organoids to study systemic diseases. For example, a liver-on-chip system analyzing increased hydrodynamic pressure in liver sinusoidal endothelial cells identified novel biomarkers for portal hypertension^396^. Emerging multi-lineage organoid systems and microfluidic devices are advancing the study of heart-liver interactions. These models mimic inter-organ signaling, crucial for understanding diseases such as cardiac amyloidosis, where liver-derived proteins like transthyretin influence cardiac function^397^. While these platforms offer dynamic insights into systemic diseases and novel therapeutic strategies, they are not without limitations^398^. A significant challenge is the lack of a universal medium, such as blood, that can supply cells with essential nutrients and growth factors across diverse organ systems.

### Precision-cut tissue slices

Precision-cut tissue slices retain the complex multicellular architecture, tissue-specific extracellular matrix, and physiological functions of the organ, and can be cultured *ex vivo* for several days^399,400^. This makes them an excellent platform for studying intercellular communication and secreted factors, such as proteins, metabolites, and extracellular vesicles (EVs).

The use of tissue slices has long been used in metabolic preclinical research. Today, advancements such as vibrating microtomes have refined the technique, enabling its application across a wide range of studies. For example, liver slices have proven as a valuable tool for investigating xenobiotic metabolism, testing anti-fibrotic drugs, and conducting toxicological studies. Importantly, slices obtained from patient biopsies allow researchers to study disease-specific pathological processes and evaluate the efficacy of pharmacological therapies^399,401^. Similarly, myocardial slices preserve cardiac structure and function, making them ideal for studying cardiac metabolism, electrophysiology, contractility, and pharmacological safety^400,402^. Slices from human failing hearts have been used to explore mechanisms of myocardial fibrosis in response to mechanical stress and to test potential anti-fibrotic treatments^403^.

By closely mimicking in vivo conditions, precision-cut tissue slices provide a robust and human-relevant system for secretome analysis. Both the tissue and the culture medium can be analyzed, enabling comprehensive profiling of secreted factors from clinically relevant tissues. For instance, the protein arylsulfatase A (ARSA) was identified as a MASLD/MASH induced hepatokine regulating systemic lysophospholipid metabolism and glycemia. Its increased secretion was first detected in the media of primary hepatocytes from a murine MASH model and later confirmed using precision-cut liver slices from healthy controls and patients with MASLD and MASH, highlighting the relevance in human disease^404^. Similarly, liver-secreted hexosaminidase A (HEXA) was identified as a hepatokine mediating liver-to-skeletal muscle communication in MAFLD. Elevated HEXA secretion was observed in the media of precision-cut liver slices from patients with MAFLD compared to controls^405^. Another study demonstrated that liver-derived EVs regulate whole-body glucose homeostasis through inter-organ signaling to skeletal muscle and the pancreas. EV secretion was enhanced in early-stage MASH, and EVs isolated from the media of precision-cut liver slices from bariatric surgery patients improved glycemic control in recipient mice, underscoring the translational potential of liver-secreted EVs^406^.

Overall, precision-cut tissue slices offer a powerful and physiologically relevant system for secretome analysis, with significant potential for discoveries that can be directly translated to clinical applications. However, a key limitation of the technique is the limited availability of viable tissue biopsies, particularly for heart tissue, which restricts the use of myocardial slices for studying cardiac-driven secretory events in heart disease.

Organoids, organ-on-chip, and precision-cut tissue systems are transformative tools for studying heart-liver interactions. As these technologies continue to evolve, they hold immense potential for the identification of the underlying mechanisms of MASLD, HFpEF and cardiometabolic disease, but also the identification of new potential targets for therapeutic intervention, paving the way for more personalized medicine and translational research.

## 6 Targeting metabolism to improve heart and liver function: mechanistic evidence

Exploring potential interactions between liver and heart, especially in the context of cardiometabolic diseases, should always aim not only at dissecting the complex underline pathophysiology: the ultimate goal is to find effective therapeutic strategies to break this axis. Several pharmacological and non-pharmacological interventions exist, targeting liver, heart or metabolic dysfunction on a systemic scale. For most, the effects on interorgan crosstalk are unknown but worth being explored.

Non-pharmacologic strategies form the foundation for managing metabolic dysfunction impacting both liver and heart health. Behavioral and environmental factors drive cardiometabolic disease by inducing oxidative stress, low-grade inflammation, IR, and lipid imbalance. TRE aligns food intake with circadian rhythms, improving glucose metabolism, reducing inflammation, and promoting weight loss^42,407,408^. TRE has been shown to enhance insulin sensitivity and reduce cardiovascular risk markers^409-411^. Whole-food-based diets like Mediterranean and DASH reduce hypertension, improve lipid profiles, and support liver function^412,413^. Dietary fiber further improves glycemic control and fosters gut microbial diversity^414^.

Exercise, another pillar of metabolic non-pharmacological interventions, enhances mitochondrial efficiency, reduces inflammation, and supports both hepatic and cardiac health^415^. Aerobic training boosts cardiovascular function, while resistance training improves glucose uptake and insulin sensitivity^416^. Stress reduction, sleep hygiene, and avoiding toxins like alcohol and tobacco further enhance metabolic balance^417-419^.

Altogether, dietary interventions and physical activity are the most effective, sustainable and safe interventions to be put in place to fight both liver and heart metabolic disease and thus reduce CVE. Despite strong evidence, barriers such as socio-economic status, time constraints, and knowledge gaps limit widespread adoption. Personalized interventions, community-based programs, and digital tools may help bridge this gap^420^.

Pharmacologic therapies provide essential support in patients with established liver or cardiac disease. Sodium-glucose co-transporter 2 inhibitors (SGLT2i), such as empagliflozin and dapagliflozin, offer dual protection. They improve cardiovascular outcomes in patients with HF (either HFrEF or HFpEF) and atherosclerotic cardiovascular disease (including acute myocardial infarction), even in the absence of T2D^421^. Similarly, SGLT2i reduced hepatic steatosis and fibrosis in MASLD, although liver-specific endpoints in non-diabetic populations remain unclear^422,423^. Glucagon-like peptide-1 receptor agonists (GLP-1 R) like semaglutide, and dual or triple incretin analogues (e.g., tirzepatide, retatrutide), reduce weight, enhance insulin sensitivity, improve exercise performance and lower risk of cardiovascular death or worsening of HF in HFpEF patients^424,425^. Semaglutide has shown steatohepatitis resolution without fibrosis improvement^426^, and ongoing trials will clarify broader hepatic benefits.

Overall, SGLT2 inhibitors and GLP-1 receptor agonists represent promising pharmacologic options for the simultaneous treatment of liver-heart metabolic disease. However, robust evidence from studies specifically designed to target multi-organ pathology—both in preclinical models and clinical settings—remains limited.

To fully understand the interorgan benefits and potential adverse interactions, metabolic interventions (potentially impacting on both liver and heart, such as non-pharmacological strategies, SGLT2i and GLP-1RA) must be rigorously tested in animal models, in vitro, and ex vivo platforms, simultaneously assessing liver and heart effects. Such interventions should be compared with organ-specific therapies (e.g. resmetirom, a thyroid hormone receptor-β agonist that improves steatohepatitis and fibrosis in patients with MASLD^427^) to test for secondary cardiac benefits. Similarly, the effects of cardiac-specific drugs should observed in the liver. Mechanistic studies should aim at dissecting molecular pathways—such as inflammation, oxidative stress, mitochondrial dysfunction, and fibrosis—involved organ-specific vs interorgan effects.

Importantly, considering the large body of evidence indicating CVE as the primary cause of mortality and morbidity in MASLD (and possibly ALD and MetALD), clinical trials designed with CVE as primary endpoints (instead of liver-related events) are needed in MASLD patients.

## 7 Conclusion

Understanding liver–heart crosstalk marks a pivotal shift in how we conceptualize CVDs—not as isolated organ dysfunctions but as an integrated, systemic syndrome. Recognizing the liver as a driver and amplifier of CVDs demands that we reframe basic research, datasets analysis, diagnostic strategies and therapeutic interventions to consider metabolic liver disease as a modifiable cardiovascular risk factor. This shift has profound implications for early screening, risk stratification, and patient management across disciplines.

The development and application of mechanistically relevant animal models and advanced in vitro and ex vivo platforms that recapitulate liver–heart interactions is critical. These systems are essential for probing interorgan signaling networks, uncovering novel molecular targets, and validating cross-organ effects of emerging therapies. Without such translational models, the causal links between hepatic pathology and cardiovascular disease remain speculative and therapeutic translation remains limited.

Interventions that target shared pathophysiological mechanisms—such as inflammation, fibrosis, lipotoxicity, and insulin resistance—hold promise for disrupting the feed-forward loops linking hepatic and cardiac dysfunction. However, most current pharmacologic trials focus on single-organ endpoints. To effectively deviate the natural history of multisystemic cardiometabolic disease, clinical trials must be intentionally designed with multi-organ outcomes in mind, including endpoints that reflect improvements in both hepatic and cardiac health.

The field stands at an inflection point: integrated approaches to research and clinical care are urgently needed to prevent and treat the growing burden of cardiometabolic disease. Collaborative efforts between hepatologists, cardiologists, internists, endocrinologists, and translational scientists will be key to unlocking therapies that reflect the biological reality of these interdependent organs. Going forward, precision medicine for cardiometabolic disease must be organ-aware, mechanism-driven, and systemically informed.

## Figures and Tables

**Figure 1:**
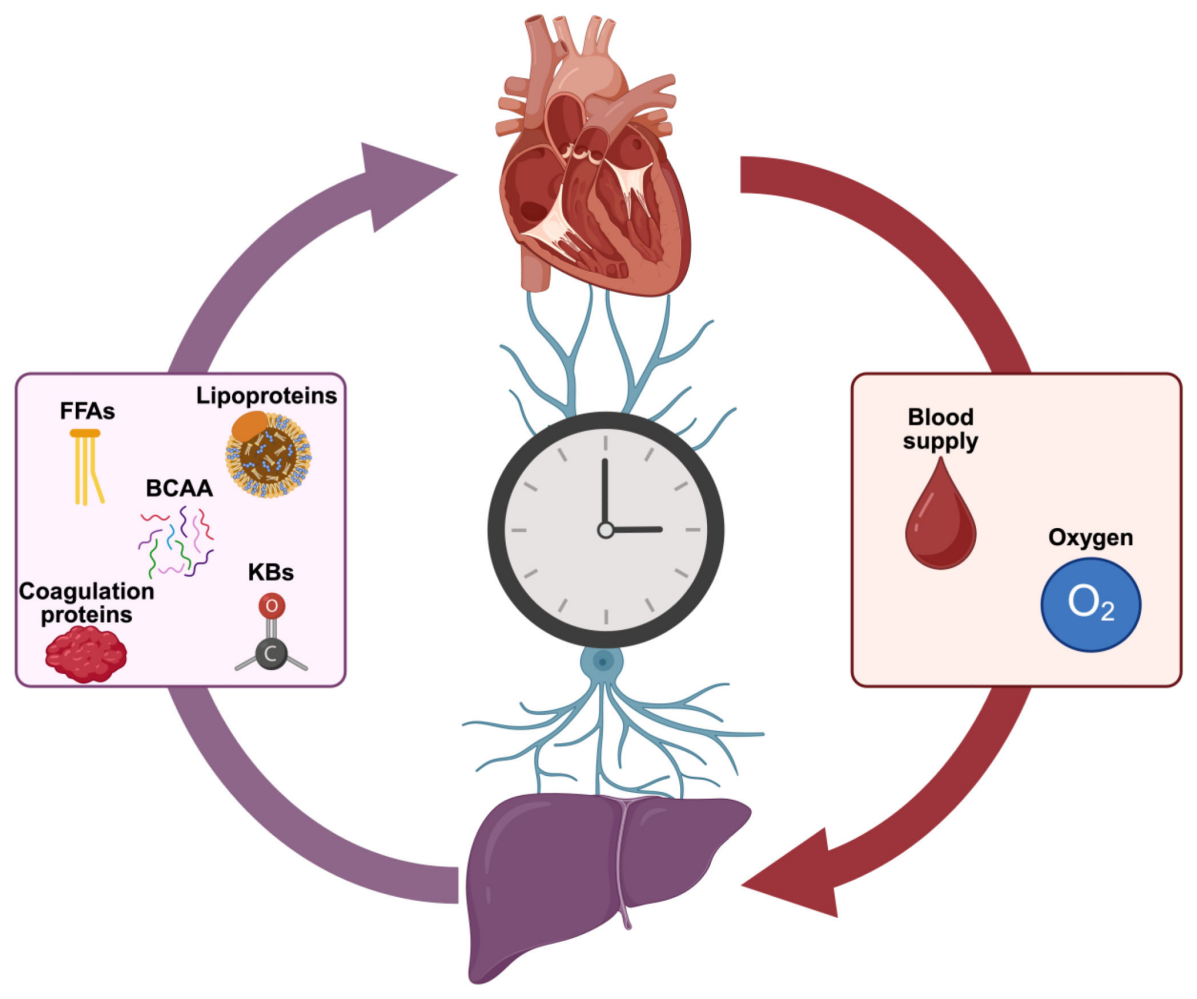
Physiological liver-heart interplay. Liver metabolism, cardiac hemodynamics, Sympathetic Nervous System (SNS) and Circadian clocks work in parallel to coordinate liver-heart homeostatic balance. *BCAA - Branched Chain Amino Acids, FFAs - Free Fatty Acids, KBs – Ketone Bodies*.

**Figure 2:**
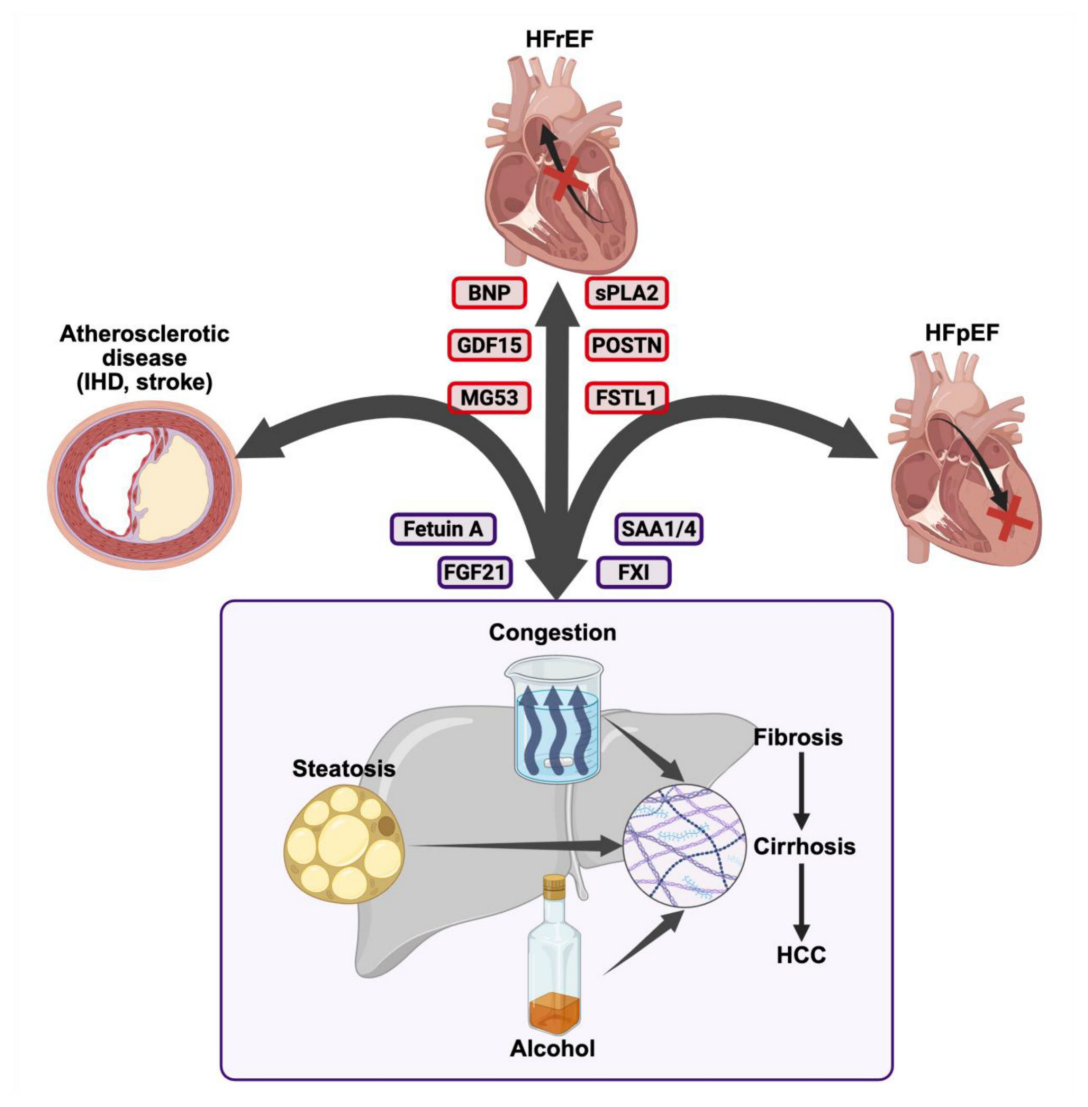
Mechanisms of pathological liver-heart interaction. Liver steatosis, alcohol consumption and congestive hepatopathy synergistically promote liver inflammation and fibrosis, eventually leading to cirrhosis and HCC. Liver disease and cardiovascular events are entangled in a pathophysiological crosstalk including hemodynamics and signaling molecules. *BNP – Brain Natriuretic Peptide, FGF21 - Fibroblast Growth Factor 21, FSTL1 - Follistatin-Like 1, FXI – Factor XI, GDF15 - Growth Differentiation Factor-15, HCC – Hepatocellular Carcinoma, HFpEF - Heart Failure with preserved Ejection Fraction, HFrEF – Heart Failure with reduced Ejection Fraction, IHD – Ischemic Heart Disease, MG53 - Mitsugumin 53, POSTN – Periostin, SAA1/A - Serum Amyloid A Proteins 1 And 4, sPLA2 – Soluble Phospholipase A*.

**Figure 3:**
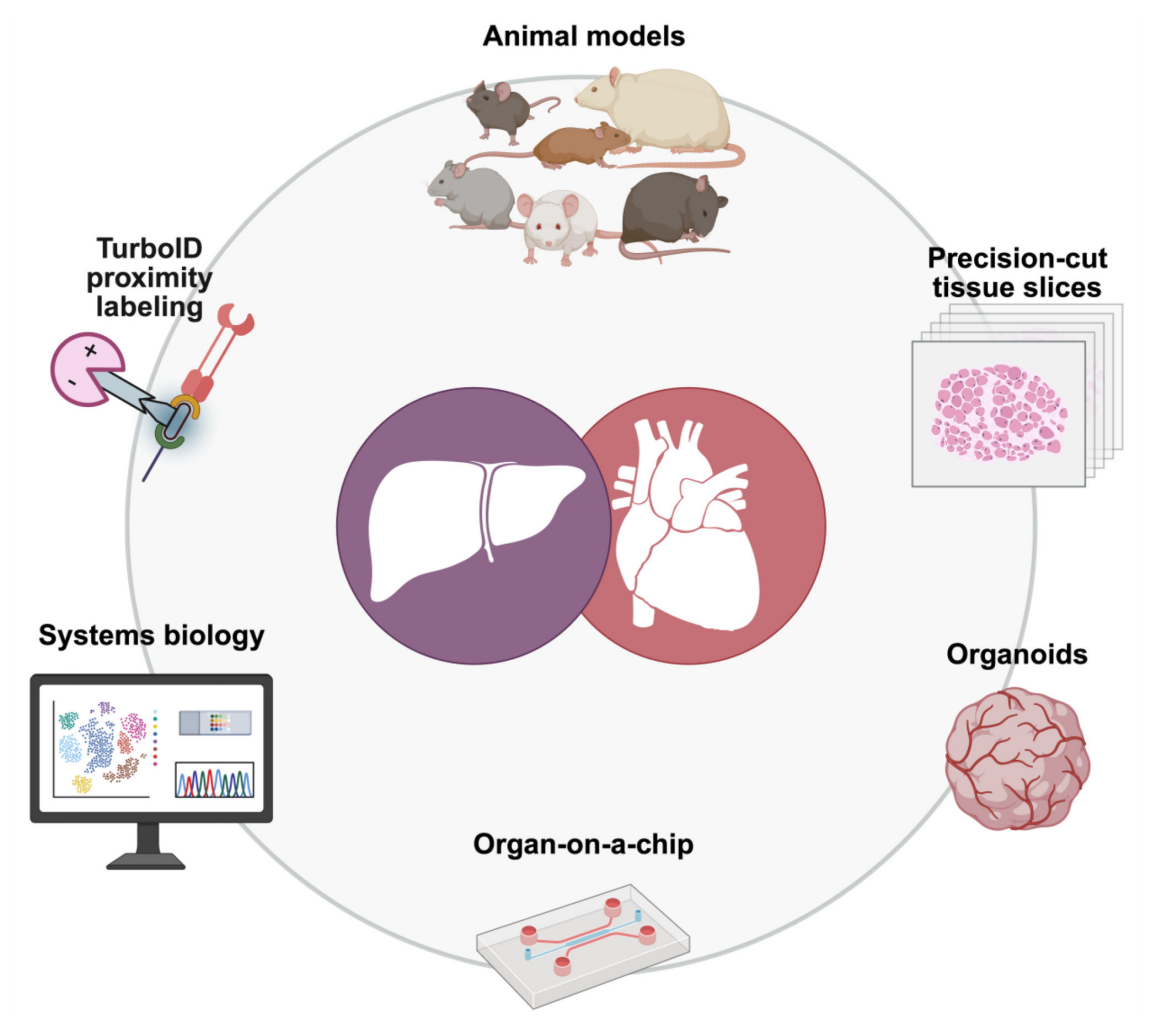
Research tools to explore cardiometabolic liver-heart crosstalk
